# How supervision and educational supports impact medical students’ preparation for future learning of endotracheal intubation skills: a non-inferiority experimental trial

**DOI:** 10.1186/s12909-021-02514-0

**Published:** 2021-02-15

**Authors:** Julian C. Manzone, Maria Mylopoulos, Charlotte Ringsted, Ryan Brydges

**Affiliations:** 1 Department of Family and Community Medicine, University of Toronto, Toronto, ON Canada; 2 Wilson Centre and Department of Pediatrics, University of Toronto, Toronto, ON Canada; 3 Centre for Health Sciences Education, Faculty of Health, Aarhus University, Aarhus, Denmark; 4 Allan Waters Family Simulation Centre and Technology-Enabled Education, St. Michael’s Hospital, Toronto, ON Canada; 5 Department of Medicine and Wilson Centre, University of Toronto, Toronto, ON Canada

**Keywords:** capaSelf-regulated learning, Self-directed learning, Lifelong learning, Theories of learning, Learning transfer, Simulation, Instructional design

## Abstract

**Background:**

Professional education cannot keep pace with the rapid advancements of knowledge in today’s society. But it can develop professionals who can. ‘Preparation for future learning’ (PFL) has been conceptualized as a form of transfer whereby learners use their previous knowledge to learn about and adaptively solve new problems. Improved PFL outcomes have been linked to instructional approaches targeting learning mechanisms similar to those associated with successful self-regulated learning (SRL). We expected training that includes evidence-based SRL-supports would be non-inferior to training with direct supervision using the outcomes of a ‘near transfer’ test, and a PFL assessment of simulated endotracheal intubation skills.

**Method:**

This study took place at the University of Toronto from October 2014 to August 2015. We randomized medical students and residents (*n* = 54) into three groups: Unsupervised, Supported; Supervised, Supported; and Unsupervised, Unsupported. Two raters scored participants’ test performances using a Global Rating Scale with strong validity evidence. We analyzed participants’ near transfer and PFL outcomes using two separate mixed effects ANCOVAs.

**Results:**

For the Unsupervised, Supported group versus the Supervised, Supported group, we found that the difference in mean scores was 0.20, with a 95% Confidence Interval (CI) of − 0.17 to 0.57, on the near transfer test, and was 0.09, with a 95% CI of − 0.28 to 0.46, on the PFL assessment. Neither mean score nor their 95% CIs exceeded the non-inferiority margin of 0.60 units. Compared to the two Supported groups, the Unsupervised, Unsupported group was non-inferior on the near transfer test (differences in mean scores were 0.02 and − 0.22). On the PFL assessment, however, the differences in mean scores were 0.38 and 0.29, and both 95% CIs crossed the non-inferiority margin.

**Conclusions:**

Training with SRL-supports was non-inferior to training with a supervisor. Both interventions appeared to impact PFL assessment outcomes positively, yet inconclusively when compared to the Unsupervised and Unsupported group, By contrast, the Unsupervised, Supported group did not score well on the near transfer test. Based on the observed sensitivity of the PFL assessment, we recommend researchers continue to study how such assessments may measure learners’ SRL outcomes  during structured learning experiences.

**Supplementary Information:**

The online version contains supplementary material available at 10.1186/s12909-021-02514-0.

## Background

Medical education cannot keep pace with the rapid advancements of knowledge in today’s society [1]. But it can develop professionals who can. Rather than continually refining curricula to integrate more content [2], medical educators could consider the type of professionals they are aiming to develop, and then use established principles from the learning sciences to design and deliver content toward that aim [3]. A concept that can inform the development of instruction and assessment methods for creating adaptive professionals has been termed “preparation for future learning” (PFL). PFL is understood as a learners’ ability to select and learn from new resources (e.g., updated guidelines, continuing education materials, colleagues, the internet) and to use that learning to facilitate solving novel problems [4]. While studies have established links between assessments of learners’ PFL outcomes and the use of certain instructional designs, only recently have researchers started to investigate which specific learning mechanisms are associated with improved PFL outcomes [5].

Where PFL has been investigated in the learning of statistics [6, 7], and in diagnostic reasoning [8], researchers have framed it as a construct that can be assessed as a type of learning transfer. That is, when learners exhibit PFL successfully, they are described as having ‘transferred in’ their previous knowledge to learn from novel resources, and as having ‘transferred out’ this new learning to solve new, related problems [9]. By contrast, assessments of ‘near transfer’ involve measuring how well learners apply their knowledge acquired in one situation (e.g., diagnosing a common respiratory condition) when performing another task with familiar surface details (e.g., diagnosing other, less common respiratory conditions) [10, 11]. Studies suggest that near transfer tests (i.e., applying knowledge to *immediately perform* a task) and PFL assessments (i.e., applying knowledge to *learn about and then perform* a novel task) represent distinct transfer outcomes [12], with PFL assessments offering greater potential to understand how instructional approaches impact learners’ capacity for future learning [6–8].

In capturing how well participants learn from new resources, studies using PFL assessments have revealed unique benefits of instructional designs such as integrated instruction [8], contrasting cases [13], and productive failure [14, 15]. An analysis of these instructional approaches suggests that most emphasize allowing learners to struggle while learning, to experiment with their own learning strategies, and to experience meaningful task variation [4, 8, 16, 17]. These characteristics align with ‘core processes’ of successful self-regulated learning (SRL): setting well-defined goals, persisting in challenging experiences requiring significant time and effort, and developing one’s own, idiosyncratic knowledge structures [18]. Evidence from medical education suggests that instruction designed to support learners to enact SRL core processes (e.g., a ‘SRL-support’, such as a list of task-specific goals from which learners can choose to set and pursue) result in improved immediate performance and retention of clinical skills [19, 20]. However, most studies have yet to clarify whether instruction including such SRL-supports also benefits learning transfer [18, 20].

By comparing different ways of supporting SRL using a PFL assessment as the primary outcome, we aim to inform the curricular mapping and assessment practices of organizations dedicated to health professions training. In particular, this may benefit schools with curricula emphasizing self-regulating, ‘master adaptive’ learners [21, 22], and accrediting bodies, such as the Liaison Committee on Medical Education [23] and Accreditation Council for Graduate Medical Education [24], that now include standards requiring explicit teaching of ‘self-directed learning’. Given the resource constraints facing most schools, we conceptualize SRL as a shared responsibility between learner and supervisor [20], which means supervisors can be present through their design of SRL-supports, rather than through direct instruction. With this perspective, we asked the question: how does a supervisor’s presence (i.e., physically present, or not), and the presence of SRL-supports (present or not) impact participants’ performance on a near transfer test and a PFL assessment?

We expected that the near transfer test and PFL assessment outcomes associated with training that includes evidence-based SRL-supports would be non-inferior to the outcomes of training that includes direct instruction from a physically present clinical supervisor. We also expected that training with either SRL-supports or Supervision would lead to improved outcomes, beyond the non-inferiority margin, compared to a training condition without either. For all comparisons, we expected that a PFL assessment would be more likely to detect larger mean group differences, relative to a near transfer test.

## Methods

### Study setting and design

This study took place at the University of Toronto from October 2014 to August 2015. The ‘humans in research’ Ethics Board approved the study; all participants provided informed consent and received a small honorarium.

**Fig. 1:**
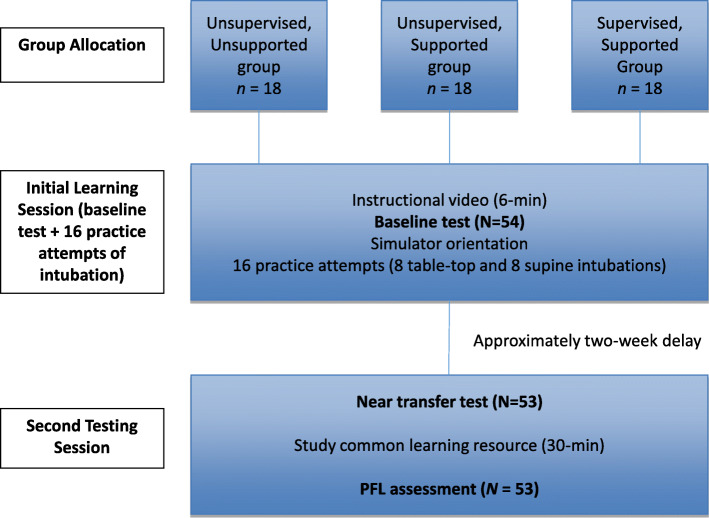
Study protocol showing participant flow across all learning sessions and assessments

In this randomized controlled non-inferiority trial [25], we considered a simulation-based training environment in which a supervisor is present as the most dominant, and likely resource-intensive, approach in health professions education (HPE). We argue that training which includes SRL-supports without requiring a supervisor’s time would be no less effective educationally, and potentially advantageous in cost- and resource-effectiveness. Our study design followed a modified double transfer protocol [6], depicted in Fig. 1.

### Participants

Via email, we recruited participants from a pool of approximately 1000 medical students and residents. We ensured participants had minimal experience performing endotracheal intubation, by setting a maximum of 10 previous successful intubations on either a patient or simulator, which our four clinician teachers came to consensus on, and is well below the 50 attempts reportedly needed for proficiency [26]. We had low response rates when recruiting from just one learner population, and consequently recruited novices from three populations: pre-clerkship students, clerkship students, and post-graduate year 1 (PGY1) Internal Medicine residents. All participants met the inclusion criteria and were randomly assigned to one of three groups, balanced by their academic year.

Based on previous studies using similar global rating scales, we expected a standard deviation of 0.70 units and set a non-inferiority margin of 0.60 units on the 5-point Likert scale [27]. We argue that 0.60 units on the GRS has been shown to be educationally meaningful in similar research studies [27], and can represent the difference between senior and junior postgraduate trainees in practice [28]. Assuming no difference between the SRL-support and Supervised conditions, we calculated that 17 participants per group would be required to be 80% sure that the lower limit of a one-sided 95% confidence interval will be above the non-inferiority limit of 0.60 [29].

### Simulated procedural skill

**Fig. 2:**
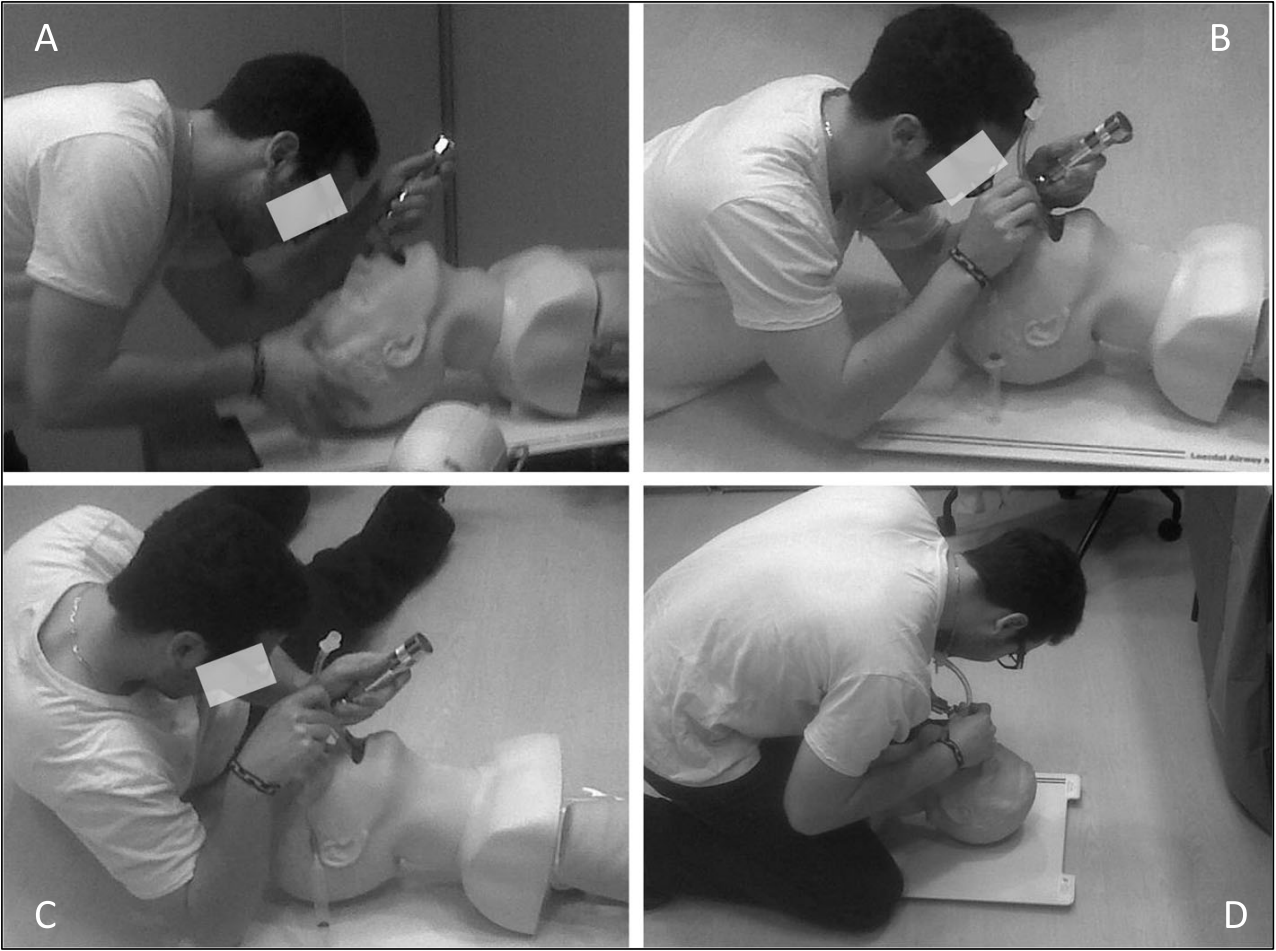
The four clinical variations of endotracheal intubation used in our study design: **a** Normal **b** Supine **c** Left Lateral Decubitus **d** Straddling

Research has consistently shown that many learning mechanisms generalize to both motor skill and verbal learning [30]. Given most research on PFL has focused on learning statistical and diagnostic reasoning concepts, we chose to extend that work to the domain of invasive procedural skills. We asked participants to perform four different variations of endotracheal intubation on the Laerdal® Airway Management Trainer: the Table-top, Supine, Left-lateral Decubitus (LLD), and Straddling positions used in our prior research (Fig. 2) [31, 32].

### Outcome measures

#### Simulated endotracheal intubation performance

We used a Global Rating Scale (GRS) developed specifically for endotracheal intubation, consisting of four subscales and an overall rating (Additional file 1). We developed this tool previously by modifying a pre-existing GRS, and we also collected favourable validity evidence in the form of strong inter-rater reliability and positive correlations with established performance metrics [31]. In the present study, we video recorded all relevant participant performances and sent them to a resident and fellow in Anesthesiology, who rated the videos independently and in a blinded fashion (i.e., unaware of participant identity, and assigned group). For each rater, we calculated the average score across the five component scales of the GRS, and then calculated an intra-class correlation coefficient (ICC) to assess inter-rater reliability. We then averaged the two raters’ GRS score, which we used in all analyses.

#### Rater orientation

During a rater orientation session, we used 12 videos selected to represent the different intubation variations. We selected example videos to represent the key GRS verbal anchors of ‘poor’, ‘competent’, and ‘clearly superior’ for each variation. The raters stopped between each video to compare ratings, discuss any disagreements, and reach consensus. We did not use the consensus scores when calculating the ICC but did include them in the remaining analyses.

### Educational interventions

We developed our three educational interventions using the proposed dimensions of SRL [20]: supervision (present or absent) and SRL-supports (present or absent). We chose to study three groups to increase the practical relevance of our research, given a fourth group (supervised, unsupported) would have been artificial (i.e., an instructor told to actively not support participants), and would likely have altered our results in favour of our hypotheses. We piloted and refined our approach for the three groups using three participants per group (data not collected during piloting).

#### Unsupervised, unsupported group

Participants assigned to this group did not receive supports beyond those provided to all three groups (i.e., an instructional video, anatomical model, notepad). Thus, participants received sufficient supports for content knowledge, but they did not receive supports for how to set learning goals, how to sequence their practice, or how to select learning strategies.

#### Unsupervised, supported group

Participants assigned to this group practiced using supports designed to help them self-regulate core-processes of SRL. The supports consisted of (all in Additional file 2): an explanation about variable and random practice schedules (i.e., alternating tabletop and supine versions randomly) to highlight the benefits of using a challenging schedule for organizing one’s practice [33, 34], a list of process goals they could set based on previous research showing that orienting learners to the processes of performance leads to better skill retention [35], and two brief interviews (conducted by author JM) that prompted participants to frame their practice in ways supportive of learning transfer [16]. In the first interview, which followed the second intubation attempt, participants reflected on how they would *replicate* their intubation approach in future experiences. In the second interview, which followed the seventh attempt, participants reflected on how they would *apply* their learning in future experiences where patient or contextual factors varied. We did not record their responses in either interview. We chose the timing of these interviews based on pilot data.

#### Supervised, supported group

Participants assigned to this group received one-on-one training with one of four university-affiliated clinician teachers. The lead author (JM) facilitated a meeting between the instructors, during which they developed a SRL-supportive teaching plan consisting of: (i) explaining key concepts for an initial 10-min, including the associated equipment, how to prepare for the procedure, and demonstrating the skill [36], (ii) organizing a blocked, variable practice schedule of the 16 attempts, with participants completing four successful attempts of Table-top variation, four successful attempts of Supine variation, and following that same sequence a second time [33], (iii) asking questions frequently, shifting from providing concurrent, hands on feedback for the first attempt to providing terminal, hands off feedback about multiple attempts, which aligns with motor-learning principles [37, 38], and (iv) debriefing participants for 10-min, asking them to verbally repeat the steps of a successful intubation [39]. We note that while some might consider this “external regulation of learning”, we consider it supportive of  SRL because participants were free to practice independently within the design set by the instructors.

### Study procedure

#### Session 1: initial session

After completing a demographic questionnaire, participants watched a six-minute instructional video outlining the steps for a successful Table-top intubation on a patient [40]. They then completed a baseline test on the simulator, performing a Supine intubation. After the baseline test, participants were oriented to the simulator, and given unlimited access to content-related educational materials: an oropharynx anatomical model, the instructional video, and a notepad and pen.

All participants experienced variable practice [33, 34], as we ensured they would perform 16 successful attempts of Table-top and Supine intubations (albeit in different sequences, depending on their assigned group). A successful attempt involved placing the endotracheal tube so both lungs could be inflated through bag-valve ventilation. The first session ended as each participant finished their 16th attempt (approximately 1–1.75 h per participant).

#### Session 2: assessing near transfer and the PFL assessment

All participants returned independently 2 weeks later. No instructors or SRL-supports were available, meaning participants experienced an unsupervised and unsupported second session, requiring them to utilize their previous knowledge and experience to regulate their learning. Participants immediately performed the left-lateral decubitus variation of intubation on the simulator. While performing this variation required participants to position their bodies differently relative to the simulator, the required technique to perform the skill was arguably familiar, which we believe fulfills the common definition of a ‘near transfer test’. [6, 11].

Next, we implemented a ‘learn-then-perform’ PFL assessment, which involves participants studying a resource containing new information, and then using that information on a subsequent performance-based assessment [8, 41]. Our participants received 30 min to read an article explaining different variations of intubation (some they had practiced, some not) [32], and to then practice these variations on the simulator. The reading was succinct and provided illustrations for six different endotracheal intubation variations. Of these six, we used the Straddling variation for the PFL assessment because this technique required significant motor skill transformations compared to those the participants learned initially (Fig. 2). By instigating such transformations, we expected that the skill would require new learning for the participants, and thus would require them to potentially demonstrate PFL: having to reconsider their equipment, to reconsider how it can be used to perform the novel approach to the procedure, and to use problem-solving processes they had not yet experienced in order to perform well in this related, yet novel condition.

### Data analysis

For the baseline, near transfer and PFL assessments, participants completed two attempts, and we averaged their GRS scores across attempts and across raters to generate a more stable estimate of performance.

Participants’ performances on the two transfer tests would not be independent; thus, we conducted separate mixed effects analyses of covariance (ANCOVAs), with baseline test as the covariate, transfer test (near and PFL) as the within-subjects factor, and either Supervision or SRL-support as the between-subjects factor. We tested whether our data violated any assumptions underlying the ANCOVA model; our inspections of normality and homogeneity of variance suggested we could proceed with our planned analyses. Further, participants’ baseline scores correlated positively with both their near transfer test scores (*r* = 0.30, *p* = 0.03) and their PFL assessment scores (*r* = 0.20, *p* = 0.16). We calculated the 95% confidence interval on the difference between group means on each test using an established procedure [42]. 

## Results

One participant from the Unsupervised, Supported group dropped out of the study. Participant demographic data are recorded in Table 1. Across all tests, we calculated an average measures ICC = 0.67, representing acceptable inter-rater reliability. All GRS data are reported below as mean ± standard deviation.

**Table 1:** Participant demographics and baseline test data

Characteristic	Unsupervised, Supported (***N*** = 17)	Supervised, Supported (***N*** = 18)	Unsupervised, Unsupported (***N*** = 18)	Statistics
Male/Female	9/8	9/9	7/11	–
Training level	Pre-clerk, *N* = 10 Clerk, *N* = 5 PGY1, *N* = 2	Pre-clerk, *N* = 10 Clerk, *N* = 6 PGY1, *N* = 2	Pre-clerk, *N* = 12 Clerk, *N* = 4 PGY1, *N* = 2	–
Intubations performed on real patients	2.12 ± 3.41	2.94 ± 4.09	1.94 ± 3.08	F_(2,50)_ = 0.41, *p* = 0.67
Intubations performed on simulators	3.71 ± 2.97	5.00 ± 4.85	5.22 ± 3.86	F_(2,50)_ = 0.73, *p* = 0.49
Intubations observed on real patients	6.82 ± 6.16	9.50 ± 10.19	8.94 ± 8.30	F_(2,50)_ = 0.49, *p* = 0.62
Intubations observed on simulators	4.29 ± 3.06	4.83 ± 3.67	5.22 ± 4.76	F_(2,50)_ = 0.25, *p* = 0.78
Self-reported motivation to learn today (0–10)	7.59 ± 1.50	7.78 ± 1.59	8.39 ± 1.46	F_(2,50)_ = 1.34, *p* = 0.27
Self-reported baseline experience (0–10);	2.18 ± 1.88	2.50 ± 1.86	2.78 ± 1.93	F_(2,50)_ = 0.44, *p* = 0.64
Baseline test performance (GRS)	2.09 ± 0.60	2.37 ± 0.91	2.14 ± 0.76	F_(2,50)_ = 0.66, *p* = 0.52

When comparing the Unsupervised, Supported group (*N* = 17, near transfer = 3.34 ± 0.68, PFL = 3.31 ± 0.66) to the Supervised, Supported group (*N* = 18, near transfer = 3.54 ± 0.45, PFL = 3.40 ± 0.64), we found that the difference in mean scores was 0.20 (95% CI of − 0.17 to 0.57) on the near transfer test, and was 0.09 (95% CI of − 0.28 to 0.46) on the PFL assessment. 

For the Unsupervised, Unsupported group, (*N* = 18, near transfer = 3.56 ± 0.53, PFL = 3.02 ± 0.74), we found that the difference in mean scores compared to the Supervised, Supported group was 0.02 (95% CI of − 0.35 to 0.39) on the near transfer test and was 0.38 (95% CI of 0.001 to 0.74) on the PFL assessment. When comparing the Unsupervised, Unsupported to the Unsupervised, Supported group, we found that the difference in mean scores was − 0.22 (95% CI of − 0.58 to 0.15) on the near transfer test and was 0.29 (95% CI of − 0.081 to 0.66) on the PFL assessment.

In summary, none of the observed differences in mean scores exceeded the 0.60 non-inferiority margin. When considering the 95% CIs, two observed comparisons did produce ranges that crossed the non-inferiority margin: both SRL-supported groups (SRL-supports alone, and with a Supervisor) compared to the Unsupervised, Unsupported group on the PFL assessment. Notably, for the near transfer test, the Unsupervised, Supported group scored lower than, and just within the non-inferiority margin, relative to the other two groups.

## Discussion

We expected that the near transfer test and PFL assessment outcomes of simulated intubation skills training with SRL-supports present would be non-inferior to the outcomes of training with direct instruction from a clinical supervisor. Our analyses showed that none of the differences in mean scores exceeded the non-inferiority margin, confirming our hypothesis. Thus, we showed that training using evidence-based SRL-supports is not inferior to the standard training using direct instruction from a clinical supervisor.

Further, we expected that a PFL assessment would be more likely to detect any differences that exceeded our chosen non-inferiority margin, relative to a near transfer test. Strictly using the non-inferiority margin, only the PFL assessment produced scores aligned with our expectations, producing 95% CIs that suggest potential, yet inconclusive benefits of training with either SRL-supports or Supervision relative to training that did not include them. For the outcomes of the near transfer test, however, we did observe that the Unsupervised, Supported group scored lower than the other two groups, and just within the non-inferiority window.

We believe the magnitude of the observed mean group differences permit discussing the modest implications of our findings for how medical educators prepare learners for future learning, for how we might assess SRL skills in HPE, and for how to refine our working definition of ‘educational support' for SRL. Below we consider how our findings might inform additional replication and extension studies.

### Sensitivity of PFL assessments to conditions supporting SRL

Similar to other studies in HPE [8], and statistics education [7], our PFL assessment produced outcomes in the direction of  our hypothesized benefits of SRL-supports and Supervision compared to when they were absent. By contrast, the near transfer test produced mixed findings that misalign with our expectations, especially suggesting non-inferiority of training that includes no SRL supports when trainees are unsupervised. Medical educators currently do not systematically measure learners’ capacity to learn from and within daily practice, and our findings suggest they may benefit from using dynamic assessments, like PFL assessments, to measure this important outcome associated with adaptive expert clinicians [21, 43, 44].

The scholars who conceptualized PFL suggested learners become better prepared for future learning after experiencing instructional designs supporting their acquisition of conceptual knowledge (i.e., knowledge about facts and principles underlying the task). Most instructional approaches studied in previous research on PFL emphasized allowing learners to fail and to experience meaningful task variation [4, 8, 16, 17]. Interpreting our findings with this lens, SRL-supports may prompt learners to experiment with using strategies they may not have otherwise considered (i.e., a form of guided discovery), which may contribute to their ability to learn and perform on the PFL assessment. Practically, our findings suggest that medical educators at institutions that have formally scheduled time for SRL into curricula will want to ensure learners receive some supports on how to regulate their learning effectively during that time, rather than leaving them both unsupervised and unsupported [20, 45]. Notably, despite the expected sensitivity of the PFL assessment, the near transfer test produced scores implying the unsupervised, supported group performed lower than the other two groups. While this finding replicates other studies suggesting near transfer and PFL assessments measure different constructs [6, 7, 15], it also suggests the selected supports for SRL could be further optimized in future studies of near transfer outcomes.

### Defining educational supports for SRL

When developing the supports for our unsupervised, supported group, we chose to use lists of process goals to focus goal-setting, multiple intubation scenarios to introduce variability during practice, interviews to focus learners on replicating and applying learned principles, and guides on how to organize practice sequences. In blending these various SRL-supports, we cannot discern how each contributed to participants’ learning outcomes. However, there is evidence in education research to suggest that a multi-dimensional approach is needed to more effectively support SRL [14, 46]. To guide future work, we propose a broad categorization of SRL-supports: content-related supports (e.g., the instructional video), practice schedule supports (e.g., organizing practice in a random sequence), and supports of core SRL processes (e.g., such as goal-setting lists).

Based on this study, we define an *educational support for SRL* as a human or material resource that guides learners to relate their learning strategies to the conceptual and/or procedural knowledge underlying the target task. Future work will inevitably refine this definition, as educators and researchers identify, implement, and test SRL-supports in their instructional designs [19]. Presently, our findings suggest learners’ future learning might benefit most through a combination of SRL-supports targeting core processes, like goal-setting, and through targeted direct supervision (e.g., helping a learner to set a study schedule, and to consider how to vary content as they study). By contrast, our findings suggest our chosen complement of SRL supports did not benefit learners’ near transfer performance.

### Limitations

Although each independent SRL-support we manipulated in the relevant experimental conditions has supporting evidence from previous studies, we acknowledge that using them in combination prevents us from clarifying the unique contributions of each to our findings. Future studies will benefit from collecting behavioural manipulation check [47] data to better understand how participants use available supports, or, alternatively, invent their own. Doing so would permit an understanding of whether results might be explained by participants’ adherence to their assigned intervention (or lack thereof) and would also permit a pragmatic understanding of which SRL-supports learners capitalize on and use.

In using intubation skills as the focus of our experimental study, we acknowledge that some of the scenarios are uncommon clinically (e.g., intubating a patient in the straddling pose). However, we chose intubation as a case given the multiple possible realistic variations of this procedure, which lends to use in a double transfer protocol. Further, we note that for this experiment, our training protocol was not designed to ensure participants developed skill mastery, and consequently note that further research is needed on how to integrate the lessons learned into practical instructional designs.

## Conclusion

Our findings suggest that providing SRL-supports (i.e., instructors or materials) during initial practice represents another educational intervention that leads to modest yet potentially educationally meaningful improvements in performance on a PFL assessment. These improvements were non-inferior compared to a Supervised training condition. Thus, our work represents the start of a conceptual link between SRL core processes and PFL assessments that we believe researchers can capitalize on further to understand the most effective ways to support learners’ SRL and to prepare them for future learning. When designing ‘self-directed learning’ time in many medical school curricula, we recommend that educators consider how they might integrate SRL-supports at the session level, using our definition above as a guide. We also challenge medical educators to consider how they can integrate PFL assessments into their programmatic assessments, which may provide a more complete picture of learners’ SRL outcomes in structured learning scenarios.

## Supplementary Information

**Additional file 1.** The Global Rating Scale (GRS) used to rate simulated endotracheal intubation performance on all tests.

**Additional file 2.** Unsupervised, supported group’s SRL-supports during session 1.

## Data Availability

The datasets used and/or analysed during the current study are available from the corresponding author on reasonable request. They have not been shared on a public database.

## Abbreviations

ANCOVA: Analysis of Covariance
ICC: Intra-class Correlation Coefficient
GRS: Global Rating Scale
HPE: Health Professions Education
LLD: Left-Lateral Decubitus
PGY: Post-Graduate Year
PFL: Preparation for Future Learning
SRL: Self-Regulated Learning
