# Supplementary material for: How supervision and educational supports impact medical students’ preparation for future learning of endotracheal intubation skills: a non-inferiority experimental trial
Source: BMC Med Educ. 2021 Feb 15;21:102. doi: 10.1186/s12909-021-02514-0 (PMC7885397; doi:10.1186/s12909-021-02514-0)
Supplement: Supplementary file 1 — Additional file 1. The Global Rating Scale (GRS) used to rate simulated endotracheal intubation performance on all tests. [file 12909_2021_2514_MOESM1_ESM.docx]

Additional File 1: The Global Rating Scale (GRS) used to rate simulated endotracheal intubation performance on all tests.

| **GLOBAL RATING SCALE OF PROCEDURAL PERFORMANCE** | | | | | |
| --- | --- | --- | --- | --- | --- |
|  |  |  |  |  |  |
| Please circle the number corresponding to the candidate's performance. | | | | | |
|  |  |  |  |  |  |
| **Respect for tissue and airway anatomy** | | | | | |
|  | **1** | **2** | **3** | **4** | **5** |
|  | Frequently used unnecessary force on airway or caused damage by inappropriate use of equipment |  | Careful handling of  airway but occasionally  caused inadvertent damage  tissue but occasionally |  | Consistently handled airway  appropriately with minimal  damage |
|  |  |  |  |  |  |
|  |  |  |  |  |  |
| **Time and motion, instrument handling** | | | | | |
|  | **1** | **2** | **3** | **4** | **5** |
|  | Many unnecessary, awkward moves, with frequent stops |  | Efficient time/motion, but some unnecessary, awkward moves |  | Clear economy of movement  and maximum efficiency |
|  |  |  |  |  |  |
| **Flow of Procedure** | | | | | |
|  | **1** | **2** | **3** | **4** | **5** |
|  | Frequently stopped  procedure and seemed  unsure of next move |  | Demonstrated some forward  planning with reasonable  progression of procedure |  | Obviously planned course of  procedure with good flow  from one move to the next |
|  |  |  |  |  |  |
|  |  |  |  |  |  |
| **Knowledge of Procedure and Equipment** | | | | | |
|  | **1** | **2** | **3** | **4** | **5** |
|  | Deficient knowledge.  Requires specific instruction for most steps of procedure and for equipment to be used |  | Knew all important steps of procedure and used appropriate equipment |  | Demonstrated great familiarity with all steps of the procedure was very familiar with equipment |
|  |  |  |  |  |  |
|  |  |  |  |  |  |
|  |  |  |  |  |  |
| **OVERALL PERFORMANCE** | | | | | |
|  | **1** | **2** | **3** | **4** | **5** |
|  | Very poor |  | Competent |  | Clearly superior |
|  |  |  |  |  |  |
