# Supplementary material for: How supervision and educational supports impact medical students’ preparation for future learning of endotracheal intubation skills: a non-inferiority experimental trial
Source: BMC Med Educ. 2021 Feb 15;21:102. doi: 10.1186/s12909-021-02514-0 (PMC7885397; doi:10.1186/s12909-021-02514-0)
Supplement: Supplementary file 2 — Additional file 2. Unsupervised, supported group’s SRL-supports during session 1. [file 12909_2021_2514_MOESM2_ESM.docx]

Additional File 2: Unsupervised, Supported Group’s SRL-Supports during Session 1

| Baseline test |  | INT |  |  |  |  |  |  |  |  | INT |  |  |  |  |  |  |  |
| --- | --- | --- | --- | --- | --- | --- | --- | --- | --- | --- | --- | --- | --- | --- | --- | --- | --- | --- |

**Applicative Knowing (i.e., knowledge that helps solve new, related problems)**

1. What features about you might make endotracheal intubation vary from case to case?
2. What features about the patient might make endotracheal intubation vary from case to case?
3. How would you modify your performance if you encounter any of that variability?

**Prompt:** What steps of the procedure can you modify and/or change if you encounter variability?

1. What steps of the procedure do you think must stay consistent?

**Replicative Knowing (i.e., knowledge of task-relevant facts)**

1. What things have you been concentrating on / thinking about during each practice attempt so far?
2. Which steps have given you trouble (e.g., forgetting a step)?
3. What specific aspects of intubation will you focus on improving in the upcoming trials?
4. How will you try to improve your technique?

**Process Goals – Available throughout**

1. Check that all equipment is available and inspect to ensure all is in working order
2. Position the patient with the Head Tilt and Chin Lift maneuver
3. Insert the laryngoscope on right side of patient’s mouth and move to it to the left with the tip of the laryngoscope at the base of the epiglottis
4. Lift the laryngoscope in the direction of the handle, doing so without pivoting the wrist
5. Insert the endotracheal tube with the right hand while visualizing the vocal cords
6. Remove the laryngoscope from the patient’s mouth and use the syringe to inflate the cuff of the endotracheal tube
7. Ventilate the patient with a bag valve mask to confirm proper placement of the tube

**Legend**

**Baseline –** Baseline

Grey boxes – single attempts

**INT -** Interview
